# Natural Compounds and Glutathione: Beyond Mere Antioxidants

**DOI:** 10.3390/antiox12071445

**Published:** 2023-07-18

**Authors:** Claudia Di Giacomo, Giuseppe Antonio Malfa, Barbara Tomasello, Simone Bianchi, Rosaria Acquaviva

**Affiliations:** 1 Department of Drug and Health Sciences, University of Catania, Viale A. Doria 6, 95125 Catania, Italy; cdigiaco@unict.it (C.D.G.); btomase@unict.it (B.T.); bianchi.simone96@gmail.com (S.B.); racquavi@unict.it (R.A.); 2 Research Centre on Nutraceuticals and Health Products (CERNUT), University of Catania, Viale A. Doria 6, 95125 Catania, Italy

**Keywords:** redox status, polyphenols, terpenoids, glucosinolates, isothiocyanates, cancer

## Abstract

The tripeptide glutathione plays important roles in many cell processes, including differentiation, proliferation, and apoptosis; in fact, disorders in glutathione homeostasis are involved both in the etiology and in the progression of several human diseases, including cancer. Natural compounds have been found to modulate glutathione levels and function beyond their role as mere antioxidants. For example, certain compounds can upregulate the expression of glutathione-related enzymes, increase the availability of cysteine, the limiting amino acid for glutathione synthesis, or directly interact with glutathione and modulate its function. These compounds may have therapeutic potential in a variety of disease states where glutathione dysregulation is a contributing factor. On the other hand, flavonoids’ potential to deplete glutathione levels could be significant for cancer treatment. Overall, while natural compounds may have potential therapeutic and/or preventive properties and may be able to increase glutathione levels, more research is needed to fully understand their mechanisms of action and their potential benefits for the prevention and treatment of several diseases. In this review, particular emphasis will be placed on phytochemical compounds belonging to the class of polyphenols, terpenoids, and glucosinolates that have an impact on glutathione-related processes, both in physiological and pathological conditions. These classes of secondary metabolites represent the most food-derived bioactive compounds that have been intensively explored and studied in the last few decades.

## 1. Introduction

Glutathione (GSH) is a tripeptide composed of cysteine, glycine, and glutamate. A French chemist, J. de Rey-Pailhade, first isolated and characterized GSH in 1888, but its composition and biosynthesis were established later [1]. Despite being discovered more than a century ago, interest in this biological molecule continues to grow, and new biological activities have been attributed to this tripeptide [2]. The reduced form, known as GSH, contains a thiol (-SH) group on the cysteine residue, which enables it to function as a reducing agent. Then, in its reduced form, GSH can donate an electron to reactive oxygen species (ROS) or other oxidizing agents, effectively neutralizing them and preventing oxidative damage to cells and tissues. GSH also plays a crucial role in enzymatic reactions that involve the reduction and detoxification of reactive compounds, such as peroxides and xenobiotics. Through its thiol group, GSH can directly react with and detoxify harmful substances, protecting cells from oxidative stress and maintaining their normal functions [3].

Furthermore, the thiol group of GSH is involved in thiol-disulfide exchange reactions, allowing GSH to regulate the redox status of proteins in the cell. This redox regulation is essential for maintaining proper protein structure and function [3]. Numerous studies have shown that GSH levels decline with age and in various diseases, including cancer, cardiovascular disease, and neurodegenerative disorders [2,3,4,5,6]. Therefore, the importance of GSH in various physiological and pathological processes has driven significant interest in understanding its role and exploring interventions to modulate GSH levels for therapeutic benefits. So, researchers have investigated strategies to replenish GSH levels in these conditions as a potential therapeutic approach [5,7,8,9,10,11]. One approach is the direct administration of GSH or its precursor molecules, such as Cysteine or N-acetylcysteine (NAC), to enhance GSH synthesis [12]. In addition to direct supplementation, research has focused on identifying natural compounds and interventions that can indirectly increase GSH levels by stimulating GSH synthesis or preventing GSH depletion. This includes the exploration of dietary interventions, such as consuming foods rich in GSH precursors or phytochemicals that enhance GSH synthesis or limit its depletion [13,14,15].

By restoring or enhancing GSH levels, it is possible to promote antioxidant defense, support detoxification processes, reduce oxidative stress, and potentially mitigate disease progression or alleviate symptoms. However, it is important to highlight that additional research is still being conducted on the therapeutic effectiveness and best methods for modifying GSH levels in particular disorders.

To completely comprehend the methods, doses, and potential side effects of GSH modulation therapies in various therapeutic scenarios, more research is required. In general, the attention paid to GSH levels in therapeutic research emphasizes the importance of this molecule in preserving cellular health and the possibility that therapies that might raise GSH levels will have a favorable effect on managing human health and disease [6]. It has been investigated whether there are any natural compounds that can enhance GSH synthesis or function. These include vitamins, minerals, and phytochemicals including polyphenols and flavonoids. These compounds have been shown to prevent oxidative stress in several cell and animal models [2,16,17,18,19]. On the other hand, over the past 15 to 20 years, evidence has emerged that GSH plays a crucial role in cell proliferation, promoting the idea that GSH depletion may enhance the efficacy of cancer treatments [8,10,20], so the role of GSH must be analyzed in light of the pathophysiology and desired outcome. In this review, particular emphasis will be placed on phytochemical compounds belonging to the class of polyphenols, terpenoids, and glucosinolates, that have an impact on glutathione-related processes, both in physiological and pathological conditions. These classes of secondary metabolites represent the most food-derived bioactive compounds that have been intensively explored and studied in the last decades.

## 2. Materials and Methods

To find literature pertinent to the study topic, a search was conducted on the PubMed, Web of Science, Scopus, and Google Scholar databases, with the publication window set to 31 May 2023. The search technique applied the following keywords: “glutathione” AND “natural compounds”; “polyphenols” AND “glutathione”; “flavonoids” AND “glutathione,” etc., in combination with terms that were relevant to the study’s goal.

The following inclusion criteria were used by the authors to determine which studies were pertinent: (1) in vitro, in vivo, and human studies; (2) original research papers and reviews published in the English language; and (3) studies focusing on the beneficial effects of natural substances on GSH homeostasis. Studies that did not fit these criteria were not considered.

## 3. Glutathione Biosynthesis and Functions

**Figure 1:**
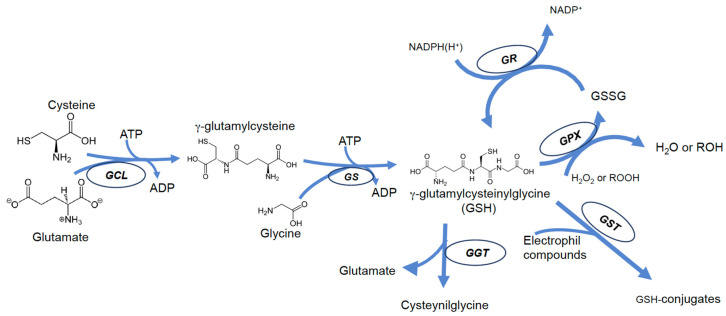
Scheme of most important pathways of GSH biosynthesis, recycling, and degradation in mammalian cells.

GSH biosynthesis is a two-step process catalyzed by γ-glutamyl-l-cysteine ligase (or γ-glutamylcysteine synthase) (γGCL, EC 6.3.2.2), and by glutathione synthase (GLS, EC 6.3.2.3). Both enzymes require ATP hydrolysis; the first enzyme (γGCL) requires ATP hydrolysis for the formation of the bond between the γ-carboxyl group of glutamate and the amino group of cysteine; the second enzyme catalyzes the addition of glycine to the dipeptide, producing GSH [1]. In contrast to the α-peptide bond, normally present in biological peptides, in the GSH molecule, glutamic acid and cysteine are bound by a γ-peptide bond. This ensures selectivity for interactions with GSH-dependent enzymes and makes the peptide resistant to hydrolysis catalyzed by typical intracellular peptidases [21,22]. The cleavage of GSH can therefore occur only by the action of γ-glutamyltranspeptidase (GGT), an enzyme exclusively found on the outer face of the membrane of some cells [23]; consequently, intracellular GSH is relatively stable, and its levels are the result of a balance with its oxidized form (GSSG) and is also related to intracellular distribution and efflux rate (Figure 1). Numerous processes, including oxidation, conjugation, and hydrolysis, deplete GSH [24].

GSSG typically accounts for 15 percent of cytoplasmic glutathione and is often considered only a byproduct of GSH metabolism. However, oxidation of GSH to GSSG may contribute significantly to its depletion under conditions of excessive exposure to ROS or RNS and may be an important event during cytotoxic agent-induced apoptosis [25,26]; if glutathione reductase (GR, EC 1.8.1.7) activity or NADPH levels are not enough to restore GSH levels by reducing GSSG back to GSH, GSH depletion occurs [27]. On the other hand, during apoptosis, mechanisms of GSH efflux from cells are activated, which further contributes to GSH depletion [28,29,30]. Thus, in this light, it is likely that all substances that can interfere in some way with one of the above mechanisms are also likely to cause disturbances in GSH homeostasis. Cells may lose GSH due to the export of its reduced, oxidized, or conjugated forms.

In addition to acting as a substrate in reactions catalyzed by glutathione peroxidase (GPX), GSH can directly react with radicals and other oxidizing chemical species [21,31,32] forming thiyl radicals that self-extinguish by reacting with each other to give GSSG [33,34,35]. In fact, oxidants such as peroxynitrite (ONOO) or hydroxyl radical (OH•) can directly oxidize GSH, leading to the production of thiyl radicals and then, of GSSG [31,36]. The reduced form of glutathione is restored by a reaction catalyzed by the enzyme glutathione reductase (GR), which uses NADPH(H+) as a reducing coenzyme [37] (Figure 1).

GSH cellular content is also dependent on mechanisms of transport; the extracellular membrane-bound enzyme GGT catalyzes the cleavage of the γ-peptide bond of both GSH and GSSG, generating cysteinyl-glycine and transferring the γ-glutamyl group onto amino acid acceptors. This event may represent a loss in glutathione.

Although all these products can be recycled and brought back in, forming GSH again according to cellular needs [23], the efflux of GSH may represent a relevant event, which can cause both an imbalance in cellular redox equilibrium and ultimately can result in oxidative stress-independent cell death [28,38]. It has been reported that GSH depletion is promoted by apoptosis produced via various stimuli (especially death receptors) [38], by activating a plasma membrane efflux transporter [27,39]. Several proteins are proposed as GSH transporters, including organic anion-transporting polypeptides (OATPs), and multidrug resistance proteins (MRPs) [27,39,40]. Some of these are ATP-dependent cotransporters and are members of the ATP-Binding Cassette (ABC) family [39,40], implicated in the sensitization of cells to apoptosis [27,28].

The potential roles of GSH are determined by its chemical structure, and its widespread presence in every living organism is evidence of its relevant biological significance.

GSH serves more purposes than just acting as an antioxidant, because it is a crucial detoxification system that plays a role in the modification of xenobiotics and2constitutes part of the process used to eliminate potentially hazardous compounds [41,42,43]. In addition, a mutual influence between estrogen and glutathione has been described [43,44,45], and involvement in the metabolism of some mediators of the inflammatory response, such as prostaglandins and leukotrienes [46,47] was reported.

Another, often overlooked, role of GSH is its involvement in the homeostasis of some metals. GSH, in fact, can interact with some metals for which the SH group shows high affinity; among these, the most studied for their impact on health are chromium, cadmium, lead, zinc, copper, and iron [21,48,49,50,51,52].

In fact, GSH plays an essential role in iron homeostasis and metabolism. The synthesis of heme, iron-sulfur (FeS) clusters and many enzymes depends on the availability of iron supplied by the labile iron pool, and iron(II)glutathione complex (FeIIGSH) is proposed to be one of the major components of it [53]. Furthermore, as heme and FeS cluster synthesis mainly occurs in mitochondria, it has been suggested that FeIIGSH can enter this organelle via di-anionic exchangers, providing a source of iron for its biosynthetic activities [54]. A connection between GSH and iron metabolism was demonstrated by the findings of Kumar et al. [55], which showed that GSH depletion induces an iron starvation-like response in yeast cells. Interestingly, a similar effect was found also with a toxic accumulation of GSH induced by overexpression of the GSH transporter Hgt1.

GSH is directly involved in FeS cluster biosynthesis, as it is necessary to transport an unidentified FeS cluster precursor from the mitochondria into the cytosol, and it is an essential cofactor for the activity of glutaredoxins [56]. What was found by Wang et al. [57] further corroborates the critical role of GSH in FeS cluster biosynthesis. In fact, they showed that as a mitochondrial carrier, namely SLC25A39, is involved in GSH import in mitochondria, depletion of this protein was directly connected with a reduction in the activity and stability of proteins containing FeS clusters.

Due to its antioxidant activity and its role in iron homeostasis, GSH can also be a potential target for cancer therapy. In fact, elevated GSH levels may be found in cancer cells as a response to the high amount of ROS produced due to their accelerated metabolism, thus protecting them from the consequent oxidative damage. Hence, strategies that lead to GSH depletion can be used to increase the efficacy of ROS-based therapies, reduce the GSH-dependent detoxification of chemotherapeutics, and induce ferroptosis in cancer cells [58]. Ferroptosis is a mechanism of cell death that occurs because of an imbalance in the intracellular redox state due to three key factors: free iron accumulation, glutathione depletion, and lipid peroxidation. GSH plays a central role in orchestrating this event since low GSH levels are linked with labile iron overload and deficiency in GPx4 activity [9]. Additionally, depletion of GSH is also able to increase the expression of heme oxygenase 1, further increasing the amount of labile iron and the consequent oxidative stress [59].

Another important feature is the intracellular distribution of GSH; although it is synthesized exclusively in the cytoplasm, it is also present in the nucleus, endoplasmic reticulum, and mitochondria, where it plays specific roles that are not merely limited to protective or antioxidant activity [60,61,62,63]. Given the prominent role played by mitochondria in oxygen consumption, ROS production, and apoptosis, mitochondrial GSH (mGSH) is of particular interest. Although the exact role played by mitochondrial GSH in apoptotic death is not yet fully understood, the control of oxidative stress in the mitochondrion involves its thiol pool, and mitochondrial GSH depletion has been shown to be closely related to the promotion of cell death [63].

Due to its dual role as a vital antioxidant and its relationship with GSH-related enzymes, GSH plays a critical role in the regulation of redox homeostasis; thus, changes in GSH levels or redox state dysregulation contribute to several illnesses and aging.

This has prompted researchers’ interest in finding therapeutic strategies to restore GSH levels by controlling the activity of GSH-related enzymes or enhancing the availability of its precursors [12,13,21].

Beyond the scope of this review, the numerous recognized antioxidant, and preventive functions of GSH are thoroughly discussed in other comprehensive papers [3,21,22].

Actually, it appears that, for the purposes of chemotherapy, the process known as glutathionylation, which results in the creation of mixed disulfides between GSH and protein thiols, is more significant than the capacity of GSH to counteract oxidative stress. In fact, oxidized protein thiol groups can undergo reactions with GSH, resulting in S-glutathionylation, a process that can be spontaneous or enzyme-driven (by glutathione S-transferases, GSTs). So, cancer cells can regulate the amount of GSH by changing the expression of enzymes involved in glutathione synthesis and metabolism [64].

## 4. GSH and Tumor Cells

Although GSH plays a prominent role in protecting against oxidative stress and, therefore, in the prevention of carcinogenesis, a growing number of studies show that tumor cells differ from normal, non-cancerous cells also in their abnormal ROS homeostatic properties. Compared to nearby non-cancerous cells, most malignant cells have a higher ROS concentration, which seems to promote growth, proliferation, metastasis, and survival in many different types of tumor cells [11,65].

To avoid ROS anticancer effects, tumor cells can alter their own antioxidant network, including pathways such as nuclear factor erythroid 2/Kelch-like ECH-associated protein 1 (NRF2/KEAP1), GSH and thioredoxin [7,8,11,64,65,66].

The primary mechanism that is triggered upon ROS generation is the NRF2 pathway. Remarkable is the frequent activation of the NRF2 pathway in many cancers, highlighting its dual function in carcinogenesis. Under normal conditions, KEAP1 collaborates with NRF2 to function. In fact, NRF2 is controlled by KEAP1, a cytoplasmic adaptor protein containing E3 ubiquitin ligases that, in non-stressed cells, binds to NRF2 motifs, causing ubiquitination and subsequently degrading NRF2 [67]. During oxidative stress, KEAP1 cysteine residues are altered, changing its conformation and interfering with its connection with NRF2. The subsequent stability of NRF2 allows it to migrate into the nucleus, where it binds to antioxidant response elements (ARE) of the genome, which activate downstream effector genes [67,68]. ARE genes activated by NFR2 include those controlling the synthesis and metabolism of GSH, antioxidant proteins such as GPX, and other drug and xenobiotic metabolizing enzymes and transporters [64].

On the other hand, NRF2’s novel function as an oncogene was demonstrated by the identification of its hyperactivation in a significant number of cancers, which gave tumor cells an advantage and led to the encouragement of growth and therapeutic resistance. The constitutive activation of NRF2 in this situation leads to the growth of the tumor as well as the progression and chemoresistance of the already-established tumor cells [11].

Increased GSH is produced as a result of the activation of the NRF2 pathway, and increased GSH levels have actually been observed in many types of cancer [69,70].

These observations seem to suggest that the hypothetical beneficial effects of antioxidant supplementation are limited or even counterproductive. In addition, since GSH plays a significant role in chemotherapy resistance, inhibiting GSH synthesis or its depletion may represent successful strategies for increasing the effectiveness of chemotherapy in light of the glutathionylation process, which, together with GST activity, contributes to the detoxification and inactivation of anticancer drugs [7,58,66,71].

## 5. Natural Compounds and GSH

### 5.1. Polyphenols and GSH

Polyphenols, a group of naturally occurring compounds widely distributed in the plant kingdom, are classified into various subclasses, including flavonoids, phenolic acids, stilbenes, and lignans. These secondary metabolites have undergone significant research to determine their mechanisms of action and potential health benefits. For instance, flavonoids, including quercetin, kaempferol, and catechins, have been linked to increased cognitive performance, a lowered risk of heart disease, and cancer prevention. Researchers have investigated the potential anti-aging and anti-inflammatory properties of resveratrol, a stilbene found in grapes and red wine [19].

Together with GSH, polyphenolic compounds play a significant role in maintaining overall health and protecting the body from oxidative stress. Interestingly, these substances have potent antioxidant effects that are comparable to GSH. They improve overall health by lowering oxidative stress and defending cells from free radical damage. According to some research, several polyphenols, including resveratrol, quercetin, and epigallocatechin gallate (EGCG), can increase the production of GSH and boost its activity in cells [30,72].

Additionally, polyphenols can help GSH levels by promoting the expression and activity of GPX, GR, and GLS enzymes, which are involved in GSH synthesis and regeneration [73]. It is crucial to remember that depending on the type of polyphenol, dosage, and individual variances in metabolism, the effects of polyphenols on GSH levels and activity may differ. A study reported that exposure of H9C2 cells to resveratrol results in an increase in GSH concentrations, GR, and GLS activities without affecting GPx activity [74]. On the other hand, treating PC12 cells with epicatechin and EGCG prevents the drop in GSH levels brought on by Pb^++^ treatment. This result can be attributed to the polyphenolic chemicals’ capacity to sustain GR activity [73].

Also, oleuropein, protocatechuic acid, and isoflavones significantly increase GR and GPx activities in J774A.1, LNCap, and PC3 cells [75].

By aiding the GSH recycling process, the various subclasses of polyphenolic compounds can contribute to the balance of GSH within cells. For instance, certain polyphenols can activate the enzyme glutamate-cysteine ligase, which oversees the initial stage in the synthesis of GSH. Polyphenols enhance body GSH levels by increasing the availability of cysteine, a crucial precursor for GSH synthesis.

Multiple investigations revealed that γGCL, a crucial gene for GSH synthesis in cells, was stimulated by relatively low quantities of flavonoids [76]. Quercetin is the most effective flavonoid, and both onion extracts, and pure flavonoids, transactivated γGCL through antioxidant response elements at the promoter level in COS-1 and HepG2 cells [76]. Structurally similar flavonoids were not as powerful; myricetin, which has just one more hydroxyl group than quercetin, was inactive, highlighting the apparent specificity of γGCL activation [77].

Although it has been suggested that one or more NRF2 binding sites (i.e., AREs/EpREs) mediate the effects of polyphenols, several flavonoid activities could be responsible for the results on the γGCL promoter. Accordingly, oxidative stress, thiol-reactive substances, and antioxidants are presumably sensitive to the release and subsequent translocation of NRF2 to the nucleus [78], which implies that the regulation of transcriptional γGCL depends on other flavonoid properties.

Various studies in vitro, in vivo, and clinical trials have documented that the protective activity of flavonoids is not only attributed to their antioxidant capacity but also to their pro-oxidant property and ability to exert modulatory effects in cells through the alteration of different signaling pathways [77,79,80,81]. Several flavonoids exhibit pro-oxidant behavior by preventing complexes I and II of the mitochondrial respiratory chain from functioning [82].

Additionally, certain flavonoids cause GSH depletion by activating the ABC transporter of the multidrug resistance protein 1 (MRP1) [83].

Since GSH depletion is the primary cause of cytotoxicity, it has long been recognized as a potential method for sensitizing cancer cells [29]. In fact, cancer cells display high levels of intracellular GSH due to an adaptive reaction to increased metabolism and consequently higher levels of ROS [84].

GSH depletion can be induced by using inhibitors of GSH synthesis such as l-buthionine sulfoximine (BSO) or through the activation of MRP1 [85,86].

In various types of neoplastic cells, including A549, HL-60, and PC-3, flavonoids can decrease intracellular levels of GSH with different effects depending on both, the structure of the compounds and the tumor cell line. The flavone chrysin induced more GSH depletion in A549 cells than in PC-3 and HL-60 at the same concentration and in a shorter treatment time. Apigenin was most effective in PC-3 cells, while hydroxychalcones and dihydroxychalcones are more active in A549 cells. The significance of a hydroxyl group at the 2′ position on chalcones was highlighted by these data, whereas a hydroxyl group at the 4′ position lessens the effect of chalcones in A549 cells and raises GSH levels in HL-60 and PC-3 cells [87]. Flavonoids′ chemopreventive abilities may be connected to their pro-oxidant behaviors. The cytotoxicity of hydroxy chalcones, apigenin, genistein, and chrysin may be caused by both interference with the mitochondrial respiratory chain and MRP-mediated GSH depletion [82,88].

Several studies in vitro highlighted that polyphenols, such as quercetin, apigenin, rhein, and resveratrol, may act in conjunction with chemotherapeutic drugs in order to increase cell cycle arrest and trigger apoptosis [72,89,90].

These synergistic effects were shown to be at least partly regulated by a decrease in GSH levels, and an increase in DNA damage was observed when polyphenols were combined with etoposide and doxorubicin [89,91,92].

Due to the broad pharmacological properties of polyphenols, it is possible to consider this large class of plant secondary metabolites as a source of complementary nutritional/pharmacological biomolecules for disease treatment and prevention strictly linked to GSH metabolism (Table 1). Although, more in-depth studies are needed on bioavailability, toxicity, and drug interactions in humans.

### 5.2. Terpenoids and GSH

Terpenoids are a diverse class of naturally occurring organic compounds widely distributed in the plant kingdom. They are responsible for the characteristic flavors and aromas of many fruits, flowers, and herbs. Terpenoids play crucial roles in plant defense mechanisms, attracting pollinators, and regulating growth and development. Terpenoids are derived from a precursor called isoprene and are classified based on the number of isoprene units they contain. Monoterpenoids, sesquiterpenoids, diterpenoids, and triterpenoids are some of the major subclasses within this chemical family. Each subclass exhibits unique chemical structures and biological activities, leading to diverse health effects [93].

Some terpenoids possess potent antioxidant and anti-inflammatory properties that can help combat oxidative stress and reduce chronic inflammation, both of which are implicated in the development of numerous diseases, including cancer [94,95,96]. Terpenoids have been found to modulate GSH homeostasis in various ways [97,98].

According to the literature, terpenoids have been reported to exhibit various anticancer effects on each stage of tumor development, acting as antiproliferative, pro-apoptotic, anti-angiogenetic, antimetastatic, and sensitizer molecules [99].

Recently, a novel form of cell death, named ferroptosis, was described in multiple diseases [100], including cancer [101]. Intracellular iron accumulation is one of the metabolic hallmarks of ferroptosis and depends on the regulation of iron trafficking across the membrane, mediated by various transporters and proteins [102,103]. Another metabolic hallmark is represented by dysregulation of the thiol redox system, composed of GSH, GR, and GPX, which, along with iron overload, resulted in the overproduction of ROS and lipid hydroperoxide (LOOH), leading to ferroptotic cell death. Notably, increasing evidence demonstrates that terpenoids induce ferroptosis in cancer [104].

Oridonin, a tetracyclic diterpenoid from *Isodon rubescens* Hemls, promotes ferroptosis through multiple effects on the thiol redox system via inhibition of GGT1 activity, GSH synthesis, and GPX4 expression in an esophageal cancer cell line [105,106]. Similarly, 18--glycyrrhetinic acid, a triterpene glycoside from medicinal herbal licorice, induces ROS/RNS production, increases lipid peroxidation, and triggers ferroptosis in MDA-MB-231 triple-negative breast cancer through downregulation of SLC7A11 expression, which negatively impacts GSH content and GPx activity [107]. Furthermore, *Betula etnensis* Raf. (Betulaceae) extract, titled betulinic acid (a pentacyclic triterpenoid), induces HO-1-mediated ferropototic cell death by reducing the GSH pool and subsequently enhancing ROS and LOOH levels [101].

Given the high levels of GSH present in most cancer cells [65], its depletion can be deleterious to cancer cells, which are thus deprived of one of the means by which they become chemoresistant, improving the therapeutic efficacy not only of ferroptotic compounds but also of ROS-based therapies (photodynamic, sonodynamic, and chemodynamic therapies) and chemotherapy [58]. Elemene, a sesquiterpene extracted from Curcuma wenyujin Y.H. Chen and C. Ling with β-elemene and trace amounts of β-caryophyllene, γ-elemene and δ-elemene isomers, is commonly used in clinical treatments of lung adenocarcinoma in combination with conventional therapy [108,109]. Its anticancer effects are linked to glutathione metabolism, which results in a decrease in the GSH/GSSG ratio and a significant reduction of SLC7A11 protein and glutaminase expression, leading to a decrease in GSH synthesis. Moreover, the low intracellular GSH concentration is also maintained by the upregulation of the glutamate-cysteine ligase modifier subunit (GCLM), the regulatory subunit of GCL, and the downregulation of GS, leading to cell apoptosis [110].

The influence of terpenoids on the glutathione network has also been studied in other pathological contexts. Recently, the protective effect of total terpenoids of Inula japonica Thunb (TTIJ) on lipopolysaccharide (LPS)-induced acute lung injury in mice was reported. TTIJs exert their beneficial effects by reducing inflammation and oxidative stress via NRF2-mediated upregulation of various genes, including glutamate-cysteine ligase catalytic subunit (GCLC) and GCLM, triggering GSH synthesis [111]. The antimigraine effects of carvacrol, a monoterpene phenol found in many aromatic plants, such as some species of Origanum [112], result from multiple mechanisms that also restore redox imbalance through an increase in GSH and GST levels [113]. In addition, glycyrrhetinic acid, in combination with paeoniflorin, a monoterpene glucoside isolated from the root of *Paeonia lactiflora* L., has shown antiparkinsonism activity in vitro and in vivo, also upregulating GCLC and GCLM [114]. The anti-diabetic properties of terpenoids have been established [115,116], and modulation of GSH metabolism seems to play a crucial role in the treatment of diabetes and its complications. For instance, the monoterpene D-limonene ameliorates diabetes in streptozotocin (STZ)-induced diabetic rats by also affecting the thiol redox system. In particular, an increase in GSH levels and GPX enzyme activity was found along with a reduction in GR activity [117]. In the same in vivo diabetic model, *Betula etnensis* Raf. ethanolic extract exerted protective activity, restoring both plasma and tissue levels of some metabolites, including GSH and lipid LOOH [118]. Intriguingly, the dysregulation of the GSH system and the induction of ferroptosis are also associated with the development of cardiovascular disease [119]. In this recent review, many strategies are proposed to activate the GSH system and alleviate the progression of myocardial injury, including the use of nutraceuticals such as terpenoids. The authors reported that britanin extracted from *Inula linearifolia* L. increased intracellular GSH levels and inhibited GPX in ferroptosis-induced myocardial I/R injury [120]. Furthermore, a water-soluble derivative of tanshinone IIA (Tan IIA), a diterpene extracted from *Salvia miltiorrhiza* Bunge, showed protective effects in an I/R-mediated myocardial injury model by triggering the GSH system via the NRF2 pathway [121].

Conversely, some terpenoids are toxic precisely because they disrupt glutathione metabolism. This is the case with the sesquiterpene lactones hymenoxon and helenalin, which showed severe hepatic toxicity in mice by causing a rapid and marked reduction in glutathione to the point of being lethal. Administration of substances that increase the intracellular glutathione pool, such as N-acetylcysteine or l-2-oxothiazolidine 4-carboxylate, protected against hepatic glutathione depletion and the lethal toxicity of both toxins [98]. Another example is the bioactivation of obacunone, which is first metabolized into a BDA intermediate by CYP3A4 and then reacts with GSH through 1,2, or 1,4 additions to form S-conjugates. The formation of these conjugates has been implicated in obacunone-induced liver injury following bioactivation reactions, as demonstrated by the presence of GSH-conjugates in the bile and urine of rats [122].

The wide range of beneficial effects that terpenoids exert on the glutathione thiol system (Table 2), coupled with some toxic effects of certain classes of these molecules, suggests that the mechanisms of action of these nutraceuticals should continue to be explored in various pathophysiological contexts.

### 5.3. Glucosinolates and GSH

In the plant kingdom, the order of Capparales includes the families of Tovariaceae, Resedaceae, Capparaceae, Moringaceae, and Brassicaceae, to which well-known crops such as broccoli, cabbage, mustard greens, etc., belong. Plants from these families are the primary source of a complex group of secondary metabolites known as glucosinolates (GSLs). These sulfur-rich molecules are essential for the plant’s defense against pathogens and pests, and recently, their potential medicinal uses and health benefits have attracted research attention [123].

GSLs (ß-thioglucoside-*N*-hydroxysulfates) are formed from an amino acid derivative, a sulfate group, and a β-d-thioglucose moiety. The chemical distinctive features of these compounds make them stable in whole plant tissues; however, when plant cells are broken or disrupted, for example, by mastication, the myrosinase enzyme, contained in the vacuole of specialized cells called myrosin cells, comes into contact with glucosinolates, causing their hydrolysis [124].

Depending on the peculiar chemical structure of the parent glucosinolate, hydrolysis of glucosinolates results in the generation of numerous breakdown products, collectively known as isothiocyanates, nitriles, and epithionitriles. Isothiocyanates (ITCs) are among those that show the most marked biological activity [123].

The potential health benefits of ITCs have been well investigated. Inhibiting the growth of cancer cells, warding off chronic illnesses such as cardiovascular problems and neurological issues, and controlling the body’s detoxification pathways are all potential benefits of these substances [125,126].

The antioxidant properties of ITCs can neutralize free radicals and prevent oxidative damage to DNA, proteins, and lipids in cells by activating the body’s natural antioxidant defense mechanisms [127]. Phase II detoxification enzymes, including GSH S-transferases and quinone reductases, which are essential for reducing reactive oxygen species and boosting cellular antioxidant capacity, have been demonstrated to be induced by glucosinolate hydrolysis products. The maintenance of cellular redox balance and defense against oxidative stress-related damage are both aided by the activation of detoxification mechanisms [128].

Following GSL hydrolysis by the myrosinase enzyme, the ITC derivatives, absorbed in the intestine, are metabolized through the mercapturic acid pathway, primarily through the conjugation with GSH by GST, with the formation of a dithiocarbamate GSH-conjugate. Some authors suggested that the ITCs’ distribution in vivo is strictly based on GSH content in the organism’s tissues [123]. Afterward, subsequent cleavage reactions catalyzed by different enzymes, such as GGT, result in the formation of the mercapturic acid derivatives, which are more water-soluble and easily eliminated in the urine and can be used as biomarkers of GSL intake [126,127,128,129]. The mercapturic acid derivatives are also found in human plasma in appreciable concentrations and seem to maintain biological activity, most likely via dissociating from the ITC derivative [128].

It is plausible that prolonged exposure to high levels of ITCs could result in a large reduction in cellular GSH since GSH conjugation is the main pathway in the metabolism of isothiocyanates. ITCs derivatives also interact with GSH by regulating its levels and activity within cells and by boosting the expression of critical enzymes involved in biosynthesis, such as γGLCL and GS [13,128,129]. This enhanced GSH synthesis and the induction of phase II detoxification enzymes aid in strengthening cellular antioxidant defenses, reducing oxidative stress, and getting rid of potentially hazardous substances [128].

The beneficial effects of ITC on GSH homeostasis were also evidenced in different animal models of metabolic disorder, where the ITC derivative of glucoraphanin, sulforaphane (SFN), boosted GSH activities and restored its production imbalance by modulating the NRF2/GPx4 pathway, which presumably is the primary mechanism, including the epigenetic, through which ITCs exert modulatory effects on GSH homeostasis [130,131,132].

ITCs have been demonstrated to possess hormetic effects on tumor growth both in vitro in different cancer cell lines such as HepG2, MDA-MB-231, and MCF-7 and in vivo. SFN at high doses suppressed tumor growth and cell proliferation, while at low concentrations, both were increased [133]. The same hormetic trend was shown in the modulation of GSH amount, suggesting that its close interaction with ITCs is strictly correlated with its antitumor and antiproliferative activities [134,135]. Such an effect can be explained since tumor cells show a high GSH content that readily interacts with elevated concentrations of ITCs, causing intracellular accumulation of GSH conjugate derivatives accountable for the antitumor activities [136].

Despite their well-documented anticancer activities (Table 3), ITCs have been found to exhibit hormetic activities in tumor cells, which involve GSH amount in a biphasic dose-response relationship, wherein low doses of these compounds can induce beneficial effects while high doses may be detrimental in different cancer models in vitro and in vivo [133,134,135].

The modulatory effects of ITCs on GSH homeostasis and its hormetic activities are significant and hold great potential for therapeutic applications. Numerous studies have demonstrated that ITCs interact directly with GSH, resulting in bioactive derivatives, and enhance its production and activity by activating the NRF2-ARE pathway, a fundamental signaling pathway involved in cellular defense against oxidative stress [130,131,132]. This activation leads to an upregulation of various enzymes involved in GSH synthesis, thereby increasing the intracellular levels of GSH [13,128].

However, further research is needed to fully understand the mechanisms underlying the modulatory effects of ITCs on GSH homeostasis.

## 6. Conclusions

A Janus-like role for GSH is emerging, whereby it may both be protective and also contribute to the resistance of cancer cells to chemotherapy. To optimize therapeutic targeting, it will be crucial to better comprehend GSH’s antioxidant-independent roles in cancer cells and to look beyond its basic antioxidant capabilities. Research into natural substances and their interactions with glutathione uncovered a fascinating and intricate interplay that goes beyond their traditional functions as simple antioxidants. Then, through its intricate interactions with various plant secondary metabolites, glutathione exhibits a broad range of cellular effects.

The studies reviewed have demonstrated that natural compounds can modulate glutathione homeostasis by stimulating key enzymes involved in its metabolism or regulating gene expression through the modulation of signal transduction pathways, affecting various cellular processes.

Although the present literature offers insightful information on the interaction between natural substances and glutathione, further study is still required to completely clarify the underlying mechanisms and maximize their potential applications.

The intricate relationships between these substances and glutathione point to the possibility of therapeutic interventions and approaches to treat many disorders, including cancer.

Moreover, it is noteworthy the importance of the emerging field of miRNAs as crucial regulatory factors of GSH metabolism, which can aggravate pathological states or favor the resolution of specific disease settings. Recently, microRNA was proposed as a novel regulation mechanism for the GSH cycle. The intracellular levels of GSH are mainly affected by miRNAs’ regulation at the level of GSH synthesis by targeting any of the proteins involved in its production. Particularly, γGLCL is regulated by various miRNAs (miR-1, miR-433) in several pathologies and experimental models [137,138]. Other studies demonstrated that miRNAs also act on GSH levels or GSH-related metabolic pathways beyond the specific targeting of the GSH enzymatic system involved in GSH synthesis [139,140,141,142]. Several groups have also examined miRNAs targeting GSH homeostasis in pathological contexts, including cancer [143], obesity and related morbidities [144], and multiple system atrophy [145].

Continued study in this field may lead to the discovery of new therapeutic strategies and an improvement in general human health.

## Figures and Tables

**Table 1:** Polyphenols’ effects on GSH metabolism.

Polyphenols	Effects on GSH Metabolism	Model	References
Resveratrol, Quercetin, EGCG	Increase GSH levels	U937 cells	[30,72]
Catechin	Increase expression of GPX, GR, and GLS enzymes	In vivo	[73]
Resveratrol	Increase GSH levels, GR, and GLS activities	H9C2 cells	[74]
Epicatechin EGCG	Prevents the drop in GSH levels	PC12 cells	[73]
Oleuropein, Protocatechuic acid, Isoflavones	Increase GR and GPx activities	J774A.1, LNCap, PC3 cells	[75]
Quercetin	Increase γGLCL activity	COS-1, HepG2 cells	[76]
Myricetin	Inactive γGLCL activity	In vitro	[77]
Flavonoids	Modulation of the mitochondrial respiratory chain of complexes I and II	In vitro	[82]
Apigenin, Naringenin, Genistein, Quercetin	Stimulate GSH transport by MRP1	In vitro	[83]
Chrysin, Apigenin	Decrease GSH levels	A549, PC-3, HL-60 cells	[87]
Hydroxychalcones	Modulation of the mitochondrial respiratory chain of complexes; MRP-mediated GSH depletion	HepG2 cells	[88]
Quercetin, Apigenin, Rhein, Resveratrol	Cell cycle arrest and apoptosis	U937, MCF7cells	[72,89,90,91,92]

**Table 2:** Terpenoids’ effects on GSH metabolism.

Terpenoids	Effects on GSH Metabolism	Model	References
Oridonin	Inhibition of GGT1 activity, GSH synthesis, and GPX4 expression; Induction of ferropototic death	TE1 cells	[105,106]
18-β-glycyrrhetinic acid	Downregulation of SLC7A11 expression; Reduction in GSH content and GPx activity; Increase in oxidative stress; Ferroptosis activation	MDA-MB-231 cells	[107]
Betulinic acid (*Betula etnensis* Raf. Extract)	Depletion of intracellular GSH; Lipoperoxidation; Upregulation of HO-1 expression; Ferroptosis induction Increase GSH in plasma and tissue; LOOH reduction	Caco-2, cells, In vivo	[101,118]
Elemene	Decrease in the GSH/GSSG ratio; Downregulation of SLC7A11, GS and glutaminase; Upregulation of GCLM	A549 and PC9 cells, In vivo	[110]
Total terpenoids of *Inula japonica* Thunb	Stimulation of GSH synthesis; Upregulation of GCLC and GCLM	In vivo	[111]
Carvacrol	Increase in GSH and GST levels	In vivo	[113]
Glycyrrhetinic acid and paeoniflorin	Upregulation of GCLC and GCLM	SH-SY5Y cells, In vivo	[114]
D-limonene	Increase in GSH levels and GPX enzyme activity; GR activity reduction	In vivo	[117]
Britanin	Enhancement of GSH levels and GPX activity	In vivo	[120]
Tanshinone IIA	Increase in GSH content and GCLC activity	H9c2 cells, In vivo	[121]
Hymenoxon and helenalin	Hepatic glutathione depletion	In vivo	[98]
Obacunone	GSH bioactivation	In vivo, Human liver microsomes	[122]

**Table 3:** Glucosinolates effects on GSH metabolism.

Glucosinolates	Effects on GSH Metabolism	Model	References
ITCs	Increase Phase II detoxification enzymes	In vitro and in vivo	[127,128]
ITCs	Decrease GSH levels	In vitro	[13,128,129]
ITCs	Increase γGLCL and GS activity	HepG2 cells	[13,130,131]
SFN	Modulation of Nrf2/GPX4 pathway	In vitro and in vivo	[130,131,132]
ITCS	Hormetic effects on cell proliferation and tumor growth through GSH levels modulation	HepG2, MDA-MB-231, MCF-7, HT1376 cells and in vivo	[133,134,135]

## Data Availability

Not applicable.
